# A suprasellar bronchogenic cyst: A case report

**DOI:** 10.1097/MD.0000000000016650

**Published:** 2019-07-26

**Authors:** Bingyang Bian, Miao Yu, Shanshan Liu, Shu Li, Ying Wei, Bei Zhang

**Affiliations:** a Department of Radiology; b Department of Pediatric Ultrasound, First Hospital of Jilin University, Changchun; c Department of Medical Imaging, Shandong Provincial Qianfoshan Hospital, Shandong University, Jinan, China.

**Keywords:** bronchogenic cysts, central nervous system cyst, diagnosis, suprasellar region

## Abstract

**Rationale:**

Bronchogenic cysts are mainly benign, congenital abnormalities, originating from the remnants of the primitive foregut. However, intracranial bronchogenic cysts have been rarely observed. Hence, better understanding of the suprasellar bronchogenic cysts is of great significance to properly perform perioperative management.

**Patient's concerns:**

A 62-year-old Chinese female was admitted to our hospital due to impairment of memory and asthenia.

**Diagnosis:**

Magnetic resonance imaging (MRI) confirmed presence of cystic lesion in the suprasellar region. The patient underwent craniotomy with resection of the cystic mass. The histopathological examinations confirmed diagnosis of bronchogenic cyst as well.

**Interventions:**

The cystic lesion was removed without complications. No drugs during follow-up were recommended.

**Outcomes:**

After discharge, the patient was advised to undergo MRI for 6 months to 1 year. No evidence of recurrence was found in the first postoperative review.

**Lessons:**

Bronchogenic cysts should be considered in differential diagnosis of cystic mass detected in the suprasellar region on MRI. Once the disease is considered, surgical resection is recommended to obtain pathological diagnosis, alleviate clinical symptoms, and prevent complications.

## 1 Introduction

Bronchogenic cysts are rare congenital anomalies of the embryonic foregut,^[1]^ and are typically confined to chest cavity, scarcely to the brain. More than 50% of bronchogenic cysts are located in the thoracic cavity. In theory, bronchogenic cysts are congenital cysts arising as an abnormal budding from primitive tracheobronchial tree.^[1,2]^

There are a variety of intracranial cystic neoplasms. The imaging manifestations of various cystic lesions may overlap, and the incidence of some lesions is extremely low. Therefore, their differential diagnosis sometimes causes a number of challenges. Importantly, identification of different kinds of intracranial cystic lesions could be advantageous to develop a reliable treatment strategy.

## 2 Case presentation

**Figure 1:**
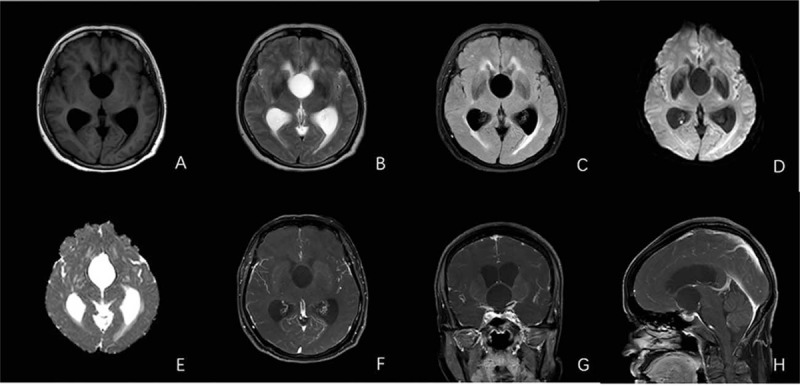
MRI of the patient's brain and pathological results. (A–E) MRI of the brain shows a cystic long T1/T2 signal intensity in the suprasellar area. (F–H) Enhanced MRI of the brain shows clear margins of the lesion and no cyst wall enhancement and internal lesion. MRI = magnetic resonance imaging

**Figure 2:**
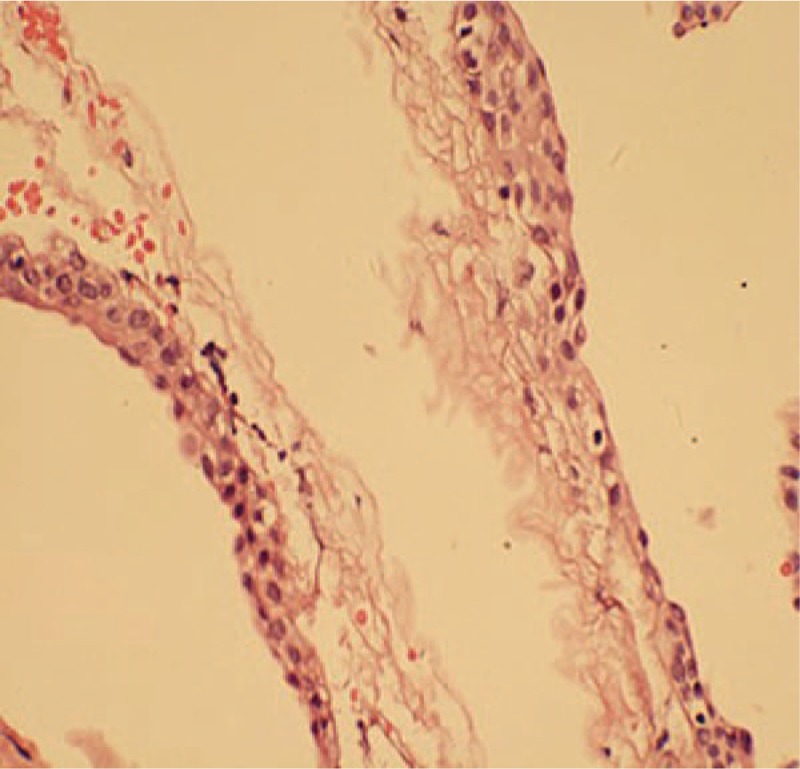
Pathological results. Pathological results indicate ciliated columnar epithelium (HE, magnification = 400×).

A 62-year-old Chinese female patient was admitted to hospital on June 20, 2018 with impairment of memory for 3 months and asthenia for a month. For the first time in March 25, 2018, the patient experienced memory decline without obvious inducement. Since May 10, 2018, the patient has developed new symptoms of general fatigue. There were occasional dizziness and headache in the course of the disease, without blurred vision, nausea, vomiting, and other discomforts. These symptoms were tolerable, thus, the patient did not receive any treatment. However, the aforementioned discomforts were not alleviated due to the onset of the disease, she therefore admitted to the hospital for treatment. The physical examination revealed that the patient was clear in consciousness. The pupil size of her both eyes was equal, and a sensitive pupillary light reflex was found. Muscular strength and muscular tone were normal as well. Physiological reflection existed and the pathological reflexes were not drawn out. No significant abnormalities were found in the parasympathetic nervous system. No diabetes, hepatitis, tuberculosis, and history of drug allergy were found in the patient. The patient had no significant medical or family history. The patient was also a primary school teacher before she retired. In order to identify the etiology, magnetic resonance imaging (MRI) of the head was undertaken on June 22, 2018. It showed cystic long T1 and long T2 signals located in suprasellar cistern (Fig. 1A and B). Fluid-attenuated inversion recovery and diffusion-weighted imaging (DWI) both showed poor signals (Fig. 1C–E), and had no transparent reinforcement screen (Fig. 1F–H). The size of the lesion was about 3.0 cm in diameter. Dilatation of bilateral ventricular and symmetrical long T1 and long T2 signals near ventricles could be observed (Fig. 1A–C). The results of complete blood count test, routine urine test, renal function, liver function, and blood biochemical parameters were all in normal level. However, the serum levels of tumor markers were not measured. The nonspecific clinical manifestations and the imaging findings could not prove the suprasellar cystic lesion. Neurosurgeon attempted to conduct surgical exploration on June 25, 2018. The neurosurgeon had working experience of more than 20 years. Supratentorial craniotomy was used under general anesthesia. Cystic lesions with clear boundary and complete capsule in suprasellar region were found during operation. After complete removal of the tumors, the capsule was peeled off and a yellowish with slightly viscous liquid was observed to flow out. Pathological findings suggested bronchogenic cysts. Respiratory epithelial cells were observed in the cyst wall (Fig. 2). In addition, immunohistochemistry was not used because of biological origin of histiocytic cells.

The patient's symptoms were remarkably relieved after surgery, and the patient and neurosurgeon were both satisfied with therapeutic effects. The patient was discharged on July 5, 2018. The patient was advised to undergo MRI every 6 months. On December 27, 2018, the patient's brain was reexamined using MRI. Postoperative changes could be observed on MR images, with no signs of recurrence or other abnormalities. According to the description of the patient, impairment of memory, asthenia, dizziness, and headache had not recurred.

## 3 Discussion

Bronchogenic cysts are mainly benign, congenital abnormalities, originating from the remnants of the primitive foregut. To date, suprasellar bronchogenic cyst has been reported only once.^[3]^ Bronchogenic cysts are rare congenital malformations, which are frequently observed in mediastinum or lung parenchyma. Although bronchogenic cysts are commonly found in the thorax,^[4]^ however, they may occur in the abdomen^[5]^ or more rarely in the suprasellar region.

Although the etiology of intracranial bronchogenic cyst is still unclear, several scholars accepted this theory. During the third week of human embryonic development, abnormal branches of the bronchial tree were severed during development of the foregut into trachea and esophagus. The severed germs migrated to the intracranial space with growth and development, resulting in intracranial bronchogenic cyst.

Unless secondary infections arise, lesions are large enough to push adjacent structures.^[6]^ Some of these bronchogenic cysts are located at atypical sites, almost leading to difficultly in differentiating them.

Ultrasound, computed tomography, and MRI are helpful to detect bronchogenic cysts.^[7]^ Nevertheless, MRI can provide more clinical information for intracranial bronchogenic cysts. The major problem in clinical trial is how to identify these diseases, such as craniopharyngioma, enterogenous cysts, arachnoid cyst, and epidermoid cyst. The craniopharyngiomas are typically cystic and solid tumors, and the cyst wall enhancement can be achieved.^[8]^ Epidermoid cysts may show diffused restriction in DWI sequences.^[9]^ However, it was difficult to differentiate arachnoid cyst from the bronchogenic cyst reported in this case. Hence, pathological examination is essential for diagnosis of bronchogenic cyst, and bronchogenic cyst can be confirmed only by histopathology.

Combining the present case with an earlier one,^[3]^ for suprasellar bronchogenic cysts, clinical practice resulted in satisfactory therapeutic effects achieved by surgery. Surgical treatment is currently one of the effective interventions for bronchogenic cysts in the chest or elsewhere. Moreover, there have been individual recurrent cases reported. A number of small embedded lesions may lead to recurrence,^[1]^ while others are due to the proximity of vital structures, leading to incomplete excision.^[10]^ A previous study reported no recurrence in a short time (6 months–1 year), which is consistent with our findings. However, incomplete excision may result in recurrent bronchogenic cyst after more than 10 years.^[11]^

Although bronchogenic cysts are typically benign tumors, however, they still have some complications, such as local infection, rupture, enlargement, and compression of surrounding organs. Besides, cystectomy should be performed to avoid recurrence due to residual cyst epithelial tissue. As the reports concerning suprasellar bronchogenic cysts are extremely rare, the clinical and imaging manifestations suffer from lack of specificity. Bronchogenic cysts should be taken into account when cystic lesions are found in the suprasellar region.

## Author contributions

Resources: Miao Yu, Shu Li, Ying Wei, Shanshan Liu.

Writing – original draft: Bingyang Bian.

Writing – review & editing: Bei Zhang.

**Resources:** Miao Yu, Shanshan Liu, Shu Li, Ying Wei.

**Writing – original draft:** Bingyang Bian.

**Writing – review & editing:** Bei Zhang.
